# Effects of aquaporin-lipid molar ratio on the permeability of an aquaporin Z-phospholipid membrane system

**DOI:** 10.1371/journal.pone.0237789

**Published:** 2020-08-18

**Authors:** Hyunki Kim, Byung Ho Lee, Moon-ki Choi, Sangjae Seo, Moon Ki Kim

**Affiliations:** 1 School of Mechanical Engineering, Sungkyunkwan University, Suwon, Republic of Korea; 2 Department of Mechanical Engineering, University of Minnesota, Minneapolis, MN, United States of America; 3 Korean Institute of Science and Technology Information, Daejeon, Republic of Korea; University of Calgary, CANADA

## Abstract

Aquaporins are water-permeable membrane-channel proteins found in biological cell membranes that selectively exclude ions and large molecules and have high water permeability, which makes them promising candidates for water desalination systems. To effectively apply the properties of aquaporins in the desalination process, many studies have been conducted on aquaporin-lipid membrane systems using phospholipids, which are the main component of cell membranes. Many parametric studies have evaluated the permeability of such systems with various aquaporin types and lipid compositions. In this study, we performed molecular dynamics simulations for four cases with different protein-lipid molar ratios (1:50, 1:75, 1:100, and 1:150) between aquaporin Z and the phospholipids, and we propose a possibility of the existence of optimal protein-lipid molar ratio to maximize water permeability. Elucidating these simulation results from a structural viewpoint suggests that there is a relationship between the permeability and changes in the hydrophobic thickness of the lipid membrane adjacent to the aquaporin as a structural parameter. The results of this study can help optimize the design of an aquaporin-lipid membrane by considering its molar ratio at an early stage of development.

## Introduction

With global population growth and climate changes, water purification techniques have gained much attention to overcome water shortages. Among the commercialized membrane-based methods for water purification, the most widely used technique is reverse osmosis (RO), which applies external pressure to a porous filtration membrane separating contaminated water and freshwater. However, an RO membrane is not applicable when the system requires desalination of high-salinity solutions and/or high recovery rates. To address this, the forward osmosis (FO) technique exploits the osmosis of highly concentrated draw solutions and pure water [1] because an FO membrane does not require external pressure to overcome the osmotic gradient. However, the FO method still demands further development to achieve high permeability for widespread use in commercialized products.

**Fig 1:**
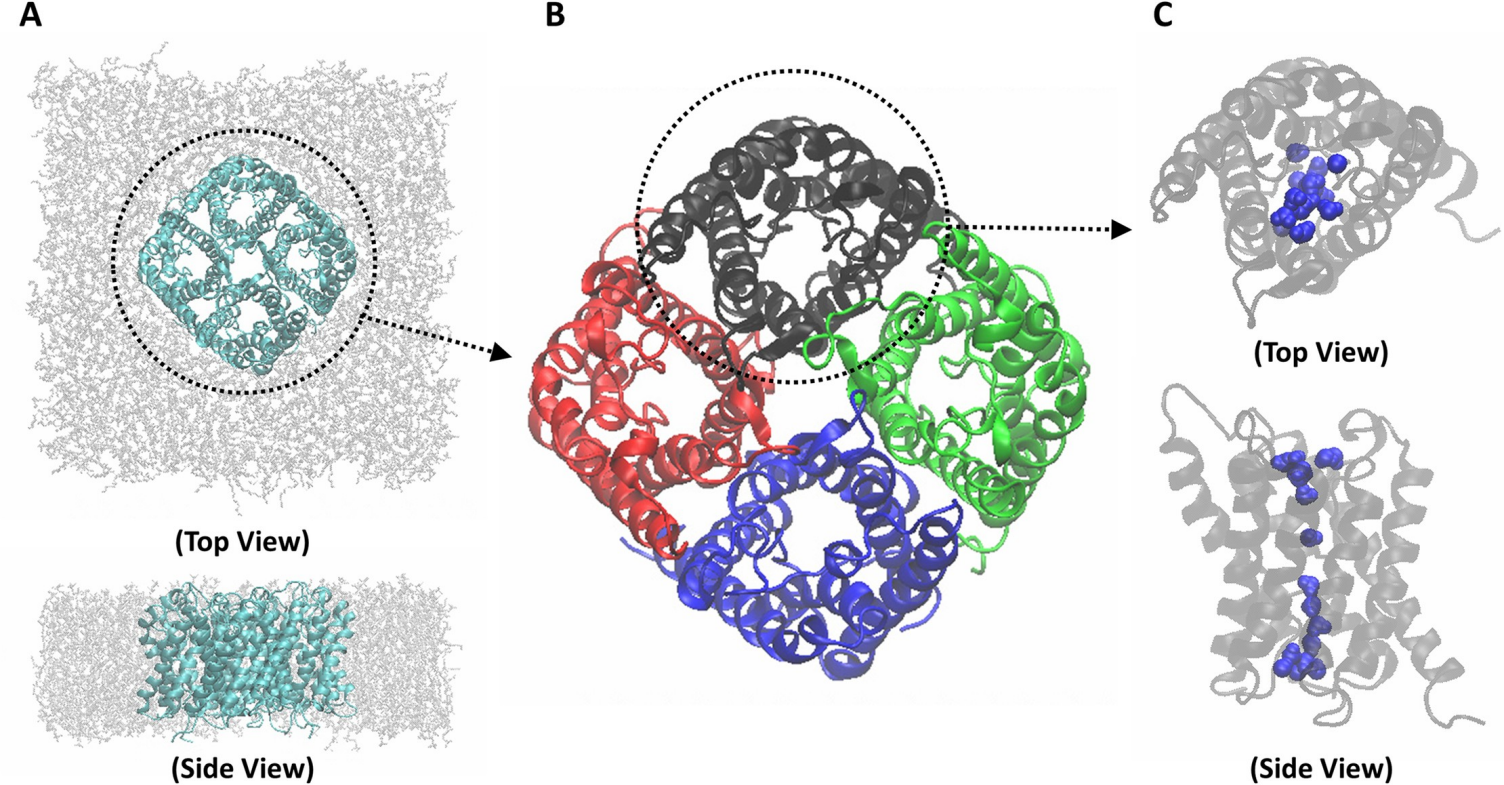
Structural schematics of an aquaporin system. (A) Aquaporin Z (AQPZ) unit tetramer (cyan) with phospholipid membrane (gray), in both top and side views. (B) Ribbon representation of a functional unit structure of AQPZ, the AQPZ tetramer in top view. The AQPZ tetramer consists of a combination of four identical monomers; each monomer is depicted in a different color. (C) Ribbon representation of an AQPZ monomer, in both top and side views. The AQPZ monomer consists of six long helixes and two short helixes, with a narrow space between the helixes that functions as a water channel. Water molecules in the channel are colored blue.

There have been many efforts to increase the permeability of FO membranes [2]. Among these, channel proteins on biological cell membranes have emerged as good candidates for FO membrane components. Especially, aquaporin-lipid membrane filtration has been attracting attention as a next-generation desalination technology owing to its high permeability. Aquaporin is a water channel protein composed of four identical monomers (Fig 1). Each monomer consists of six long helixes and two short helixes, and water transports through the central area of the helixes. The unique structure of the water channel of each monomer allows high water permeability (i.e., on the order of 10^9^ molecules/s). Jung *et al*. [3] suggested an hourglass model that describes the narrow central structure of aquaporin, and Tang and Kim [4] confirmed with molecular dynamics (MD) simulations that the high water permeability of aquaporin is caused by its hourglass structure. In addition, aquaporin selectively transports water molecules [5–7]. Aquaporin Z (AQPZ) is present in the cell membrane of *Escherichia coli* (*E*. *coli*) and is suitable for commercialized systems. However, in order to use an aquaporin desalination system at the industrial level, the filtration design of the system must be optimized.

**Fig 2:**
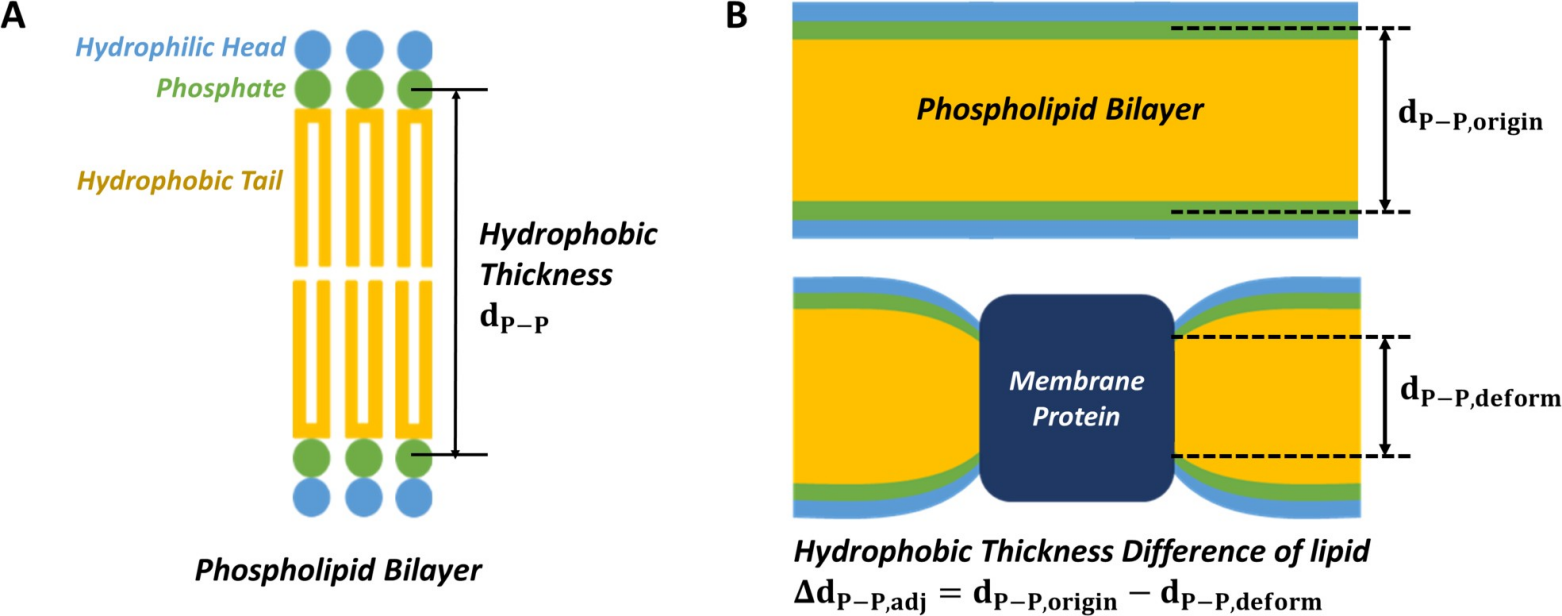
Schematics of a phospholipid bilayer and hydrophobic thickness difference of a phospholipid bilayer. (A) A phospholipid consists of a hydrophilic head (light blue), phosphate (green), and hydrophobic tails (yellow). The thickness of the hydrophobic part is called the hydrophobic thickness. The hydrophobic thickness of the lipid is the same as the phosphorous atom-to-phosphorous atom distance (d_P−P_). (B) When a membrane protein (dark blue) is induced into a phospholipid bilayer, the hydrophobic thickness difference of the lipid, Δd_P−P,adj_, is defined as the difference between the original hydrophobic thickness, d_P−P,origin_, and the hydrophobic thickness after deformation d_P−P,deform_.

Kumar *et al*. [8] experimentally examined an aquaporin-ABA copolymer membrane system to verify the effects of aquaporin-copolymer ratios on permeability, and Tong *et al*. [9,10] studied an aquaporin-phospholipid membrane system to investigate the effects of composition, cholesterol content, and the hydrocarbon length of lipids on permeability. Mobashery *et al*. [11] demonstrated that the function of a gramicidin channel is affected by hydrophobic mismatches, and Kim *et al*. [12] analyzed this effect with an MD simulation. Garavagila *et al*. [13] determined the effects of hydrophobic thickness on the ion permeability of a KscA ion channel. The structural changes in lipid membranes surrounding membrane channels have thus been shown to be significant factors in determining their functionality, and hydrophobic thickness is a key structural parameter for lipids. (Fig 2) Computational analyses like MD simulation can reveal the influence of design parameters on water permeability in the early stage of aquaporin filtration system design, such as selecting the type of aquaporin and the composition of the membrane component.

In this study, we introduce a prediction method for optimal aquaporin-lipid molar ratios for water permeability, with a short production simulation. This method can successfully and effectively predict membrane permeability using only the structural parameters that represent the degree to which the lipid bilayer and aquaporin are stressed (changes in the hydrophobic thickness of the lipid bilayer). Our results show the effect of the protein-lipid molar ratio on the osmotic permeability, as well as the existence of an optimal molar ratio. Our method reduces the computational cost of permeability prediction to less than one-tenth that of a direct calculation case and enables the simulation-based design of an optimal membrane system at an early stage of filtration system development.

## Materials and method

### System preparation

**Fig 3:**
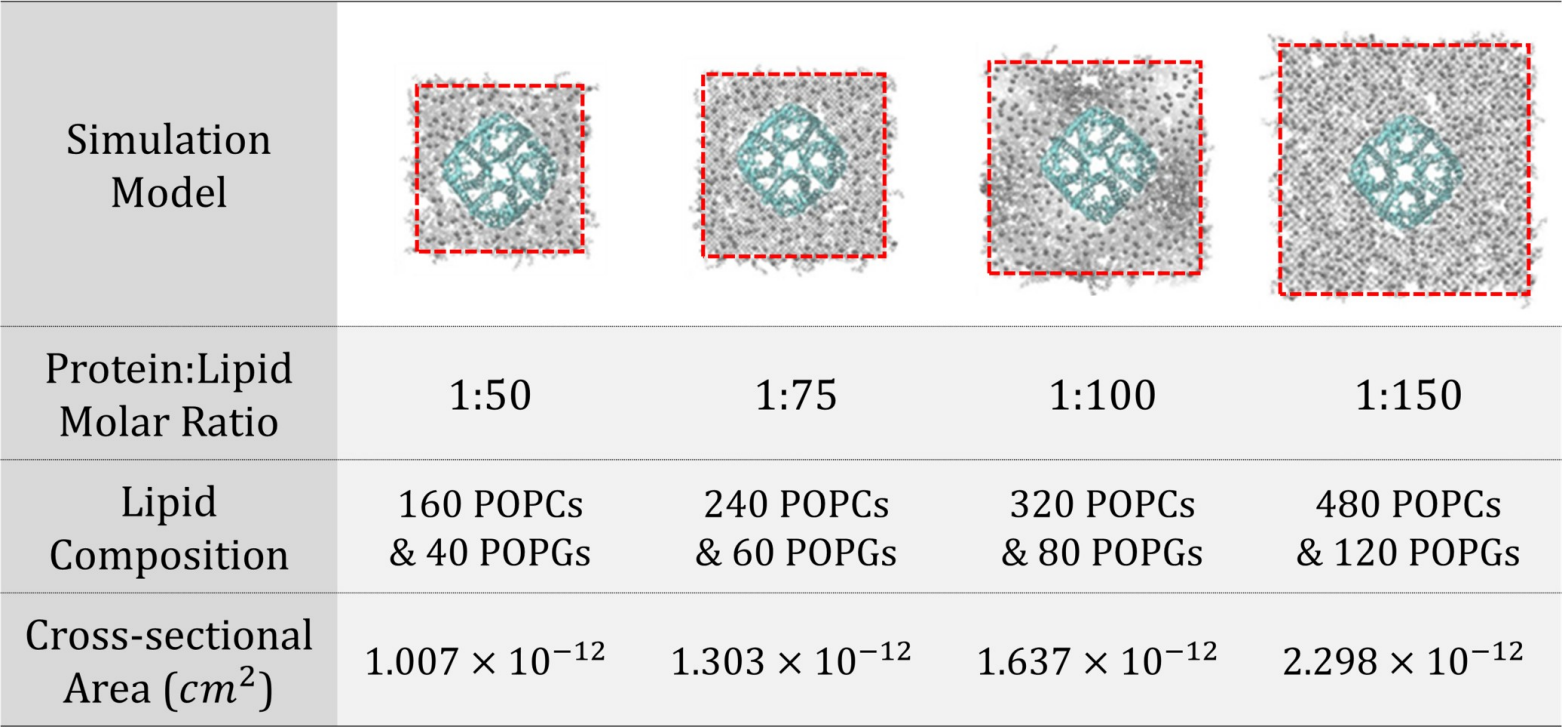
Molecular dynamics simulation models. Four models were constructed with different protein-lipid molar ratios (1:50, 1:75, 1:100, 1:150) between the proteins (cyan) and lipids (gray), and a periodic boundary condition in all directions (noted with red dashed lines). Water molecules and ions are not shown for clarity. The lipid component of the system consists of 1-palmitoyl-2-oleoyl-sn-glycero-3-phosphocholine (POPC) and 1-palmitoyl-2-oleoyl-sn-glycero-3-phosphoglycerol (POPG) in a ratio of 8:2. The cross-sectional area of the system which was proportional to the channel direction was calculated.

We constructed a system using an AQPZ unit tetramer to verify the permeability performance of a functional unit of AQPZ on a lipid membrane. The membrane systems consist of AQPZ (PDB code: 1RC2) [14] and lipids. Here, lipids are composed of 1-palmitoyl-2-oleoyl-sn-glycero-3-phosphocholine (POPC), and 1-palmitoyl-2-oleoyl-sn-glycero-3-phosphoglycerol (POPG) in the ratio of 8:2. Although both POPC and POPG have similar structures, POPC is an uncharged lipid but POPG is a negatively charged one. By mixing POPC and POPG, the stability of the unilamellar proteoliposome increases, and the aggregation between proteoliposomes decreases [10]. Aggregation does not occur on the scale of MD simulation, but for consistency with the lipid system used in experimental studies, the mixture of phosphatidylcholine and phosphatidylglycerol was adopted as a model. We constructed the system using a CHARMM-GUI membrane builder [15–19]. The hydration number was 65 or greater, which is suitable for lipid membrane simulations. We added 0.15 M sodium and chloride ions to neutralize the systems. Fig 3 lists the four aquaporin-lipid membrane models, which contained protein-lipid molar ratios of 1:50, 1:75, 1:100, and 1:150.

### Simulation protocol

All simulations were conducted on NAMD v2.10 with CUDA and a CHARMM36 force field [20–23]. Van der Waals forces were calculated between atoms at a distance of 10 Å and were smoothly reduced to zero at 12 Å. An atom pair list was made within 16 Å for every 10 time steps. All bonds involving a hydrogen atom were regarded as rigid. The particle mesh Ewald (PME) method was applied to evaluate full electrostatics. The direct space tolerance of the PME method was 10^−6^, the interpolation order was 6, and the maximum distance between the PME grid points was 1.0 Å. Equilibration simulations were conducted according to the protocol suggested by Jo *et al*. [19]. An initial constrained simulation was conducted for 25 ps with harmonic restraints of force constants from 0.1 to 10 kcal/(mol·Å^2^), which is suggested by Jo *et al*. [19].

Subsequently, equilibration simulations without harmonic restraints were performed for 10 ns. The root-mean-square deviation of the aquaporin protein and system area of the model fully converged after 10 ns, indicating that the systems were sufficiently equilibrated. Also, as shown in the study of Hong C. *et al*. [24], heterogeneous lipid membranes created by CHARMM-GUI [15–19] have very low autocorrelation of radial distribution function initially and then it disappears quickly as the simulation runs. It implies that the proposed lipid membranes with POPC and POPG are fully mixed up through the 10 ns equilibration simulation. Production simulation was performed for 50 ns and was repeated four times for each case to calculate system osmotic permeability. During the equilibration simulations, the time step of the simulation was 1 fs/step for its stability, and time steps for the production run were set to 2 fs/step. In the equilibration simulations, velocity rescaling with 500 time steps was used to maintain the temperature at 303.15 K. The Langevin dynamics was applied to keep the temperature constant at 303.15 K. Its damping coefficient was 1 ps-1. Since the Langevin thermostat, which uses velocity rescaling, was used to maintain the temperature, a lower diffusivity can be derived compared to that from the microcanonical ensemble. However, for each case, the simulation was performed using the same frequency of velocity rescaling, so there is no limitation in the comparison between cases. Although the problem of low diffusivity could be overcome by using the Nosé-Hoover thermostat, such simulation is nonergodic. Because of this, we used the Langevin thermostat to allow an ergodic simulation. The system pressure was kept constant at 1 atm using the modified Nosé-Hoover method with the Langevin dynamics [25,26]. The barostat oscillation and damping time scale were 50.0 fs and 25.0 fs, respectively. The simulation boxes were allowed to fluctuate independently but the x-y plane was restrained to a constant ratio.

### Permeability calculation

**Fig 4:**
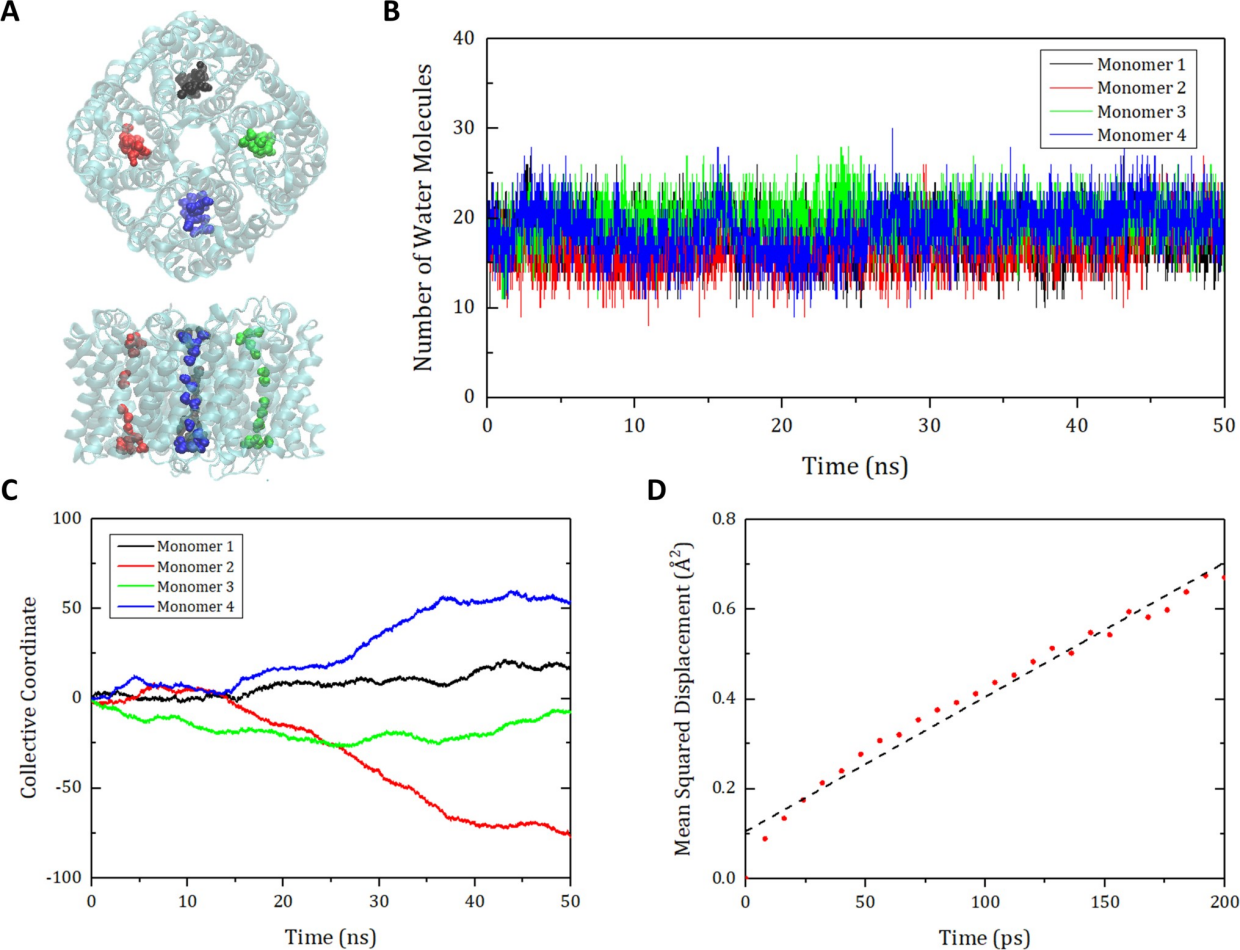
Schematics for calculating the osmotic permeability of aquaporin with the collective coordinate model [27]. (A) Schematics of aquaporin Z (AQPZ) and water molecules in the channel area. (B) Number of water molecules in the monomer channels. (C) Collective coordinates for each monomer channel of the AQPZ along 50 ns of the MD simulation. In (A)~(C), the four monomer channels are depicted with different colors (black, red, green, and blue). (D) Mean squared displacement (MSD) for the collective coordinates of the monomer channels of the AQPZ. Each collective coordinate of the four channels is divided into trajectories for the same time interval of 200 ps. Then the MSD is calculated by regarding 1,000 trajectories as 1D random walks performed 1,000 times. Linear regression (dashed line) is applied to derive the slope, which is equal to twice the diffusivity. The osmotic permeability is proportional to this diffusivity.

To calculate the osmotic permeability of the aquaporin tetramer unit (*p*_*u*,*tet*_), we used a collective coordinate model [27]. As depicted in Fig 4, the positions of the water molecules in the four channels in a tetramer were extracted and a collective coordinate *n*(*t*) was calculated using the following equation:

$$dn=\sum_{i\in s(t)}\frac{dz_{i}}{L}$$ (1)

where *s*(*t*) is the set of water molecules in a channel at time *t*, *dz*_*i*_ is the displacement of a water molecule *i* for *dt* in the direction *z* as the channel longitudinal direction, and *L* is the channel length [27]. Because the water molecules in the aquaporin channel move in the z-direction, the collective coordinate *n*(*t*) is considered as a 1D random walk that satisfies the Einstein relation 〈*n*^2^(*t*)〉 = 2*D*_*n*_*t*, where 〈*n*^2^(*t*)〉 is the mean squared displacement (MSD) of *n*(*t*), and *D*_*n*_ is the diffusion coefficient of *n*(*t*). Each collective coordinate of the four monomer channels was divided into trajectories for the identical time interval of 200 ps. Then the MSD was calculated by interpreting 1,000 trajectories as 1D random walks performed 1,000 times. To derive the diffusivity from the Einstein relation, the slope of the MSD was calculated by linear regression using the least-squares method. Then the osmotic permeability of the aquaporin monomer *p*_*u*,*mon*_ was derived through *p*_*u*,*mon*_ = *v*_*w*_*D*_*n*_, where *v*_*w*_ is the average volume of a water molecule. Finally, the osmotic permeability of the aquaporin unit tetramer *p*_*u*,*tet*_ was calculated by *p*_*u*,*tet*_ = 4×*p*_*u*,*mon*_.

### Hydrophobic thickness calculation

The hydrophobic thickness of the lipid membrane was calculated by measuring the phosphorous atom-to-phosphorous atom distance (d_P−P_) (Fig 2A). The hydrophobic thickness differences of lipid membrane adjacent to protein (Δd_P−P,adi_) indicated a difference between the original d_P−P_ (d_P−P,origin_) and the deformed d_P−P_ (d_P−P,deform_), which were obtained from the lipids not including a channel protein, and contiguous to the protein, respectively (*i*.*e*., Δd_P−P,adj_ = d_P−P,origin_−d_P−P,deform_ in Fig 2B). The representative value of Δd_P−P,adj_ of one case was the average of the five values calculated every nanosecond from the initial 5-ns production simulation.

## Results and discussion

### Protein-lipid molar ratio and permeability

**Fig 5:**
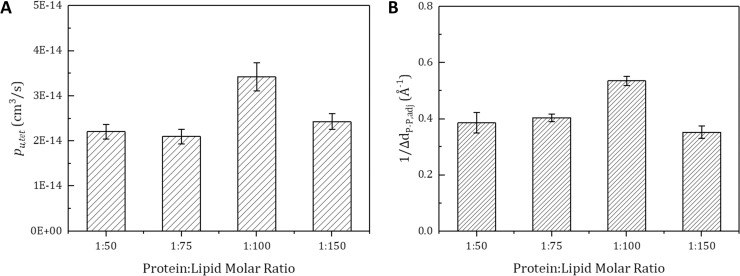
Comparison between calculated osmotic permeability of the unit tetramer (*p*_*u*,*tet*_) and predicted trend based on hydrophobic thickness difference. (A) The *p*_*u*,*tet*_ value for each protein-lipid molar ratio is calculated from a whole production simulation of four repetitive production simulations (50 ns each). (B) Δd_P−P,adj_ is defined as the difference between the original hydrophobic thickness and the hydrophobic thickness of the lipid membrane contiguous to the membrane protein. The Δd_P−P,adj_ value of each protein-lipid molar ratio is calculated by averaging four repetitive simulations (5 ns each). It was predicted that *p*_*u*,*tet*_ would be inversely proportional to Δd_P−P,adj_ (*p*_*u*,*tet*_ ∝ 1/Δd_P−P,adj_) because a large amount of stress caused by a large Δd_P−P,adj_ value would hinder the aquaporin’s function. The error bars indicate standard errors. The Pearson correlation coefficient between *p*_*u*,*tet*_ and 1/Δd_P−P,adj_ is 0.878, and the p-value is 0.00185 at the 5% significance level in a two-sided test.

To find the optimal conditions of the osmotic permeability of an aquaporin tetramer (*p*_*u*,*tet*_), we calculated the unit tetramer permeability using the collective coordinate model and a production simulation of 50 ns. As can be seen in Fig 5A, the protein-lipid molar ratio 1:100 case produced the highest *p*_*u*,*tet*_, which was about 40% higher than the other cases; it was followed by the 1:150, 1:50, and 1:75 cases. This result suggests that the molar ratio of proteins and lipids is a very important factor in determining water permeability.

### Prediction through hydrophobic thickness

To further analyze the difference of the permeability of aquaporin with respect to the protein-lipid molar ratio, the hydrophobic thickness difference (Δd_P−P,adj_) was calculated. When a membrane protein is inserted into a lipid membrane, the difference between the hydrophobic thickness of the membrane protein and the hydrophobic thickness of the lipid membrane adjacent to the membrane protein makes the thickness of membrane deformed to become closer to the hydrophobic thickness of the membrane protein. The higher the value of Δd_P−P,adj_, the greater the deformation of the lipid membrane that occurs in the region adjacent to the aquaporin; this decreases the flexibility of lipid membrane, thereby decreasing the membrane protein’s function. Therefore, we regard the inverse of the Δd_P−P.adj_ value as the relative degree of the aquaporin’s function, which is the aquaporin permeability of each molar ratio case.

As can be seen in Fig 5B, the protein-lipid molar ratio 1:100 case produced the lowest Δd_P−P,adj_ value, followed by the 1:75, 1:50, and 1:150 cases. As shown in Fig 5, the results show that the inverses of the Δd_P−P,adj_ and *p*_*u*,*tet*_ values are quite similar to each other. The Pearson correlation coefficient between them is 0.878, and the *p*-value is 0.00185 at the 5% significance level. Both graphs show that the 1:100 case has the highest water permeability. As can be seen from the result, the trend in permeability for protein-lipid molar ratio is not monotonic and that can be predicted with the Δd_P−P,adj_. And the relationship between the permeability and Δd_P−P,adj_ can be interpreted from the viewpoint that the stress applied to the membrane protein is lowered at a particular molar ratio and can be predicted based on Δd_P−P,adj_. Mechanically, if the protein is inserted in the lipid membrane, the membrane adjacent to the protein is compressed. But, the membrane in a little farther region swells slightly for force equilibrium. Therefore, in a specific protein-lipid molar ratio, compression from the protein and tension from the adjacent protein are applied simultaneously at the lipid membrane in the vicinity of one protein, resulting in a smaller degree of membrane compression. By this principle, in the case of protein-lipid molar ratio 1:100, the overlap of lipid membrane changes by the insertion of membrane proteins made Δd_P−P,adj_ smaller, which made the aquaporin less stressed and natural. And in the case of protein-lipid molar ratio 1:150, the distance between the adjacent membrane proteins is farther than that of protein-lipid molar ratio 1:100, so the influence of the insertion of an adjacent protein is reduced. Therefore, the protein-lipid molar ratio 1:100 case has a smaller Δd_P−P,adj_ and higher *p*_*u*,*tet*_ than the protein-lipid molar ratio 1:150 case. Like the aquaporin-lipid membrane system, similar results can be found in the aquaporin-ABA copolymer system. Kumar *et al*. [8] reported through an experimental study that a certain aquaporin-polymer molar ratio exists that optimizes permeability. Consequently, this simulation result validates that we can predict the tendency of *p*_*u*,*tet*_ with a structural parameter, Δd_P−P,adj_, from a short production simulation of 5 ns.

### Prediction of system level permeability

**Fig 6:**
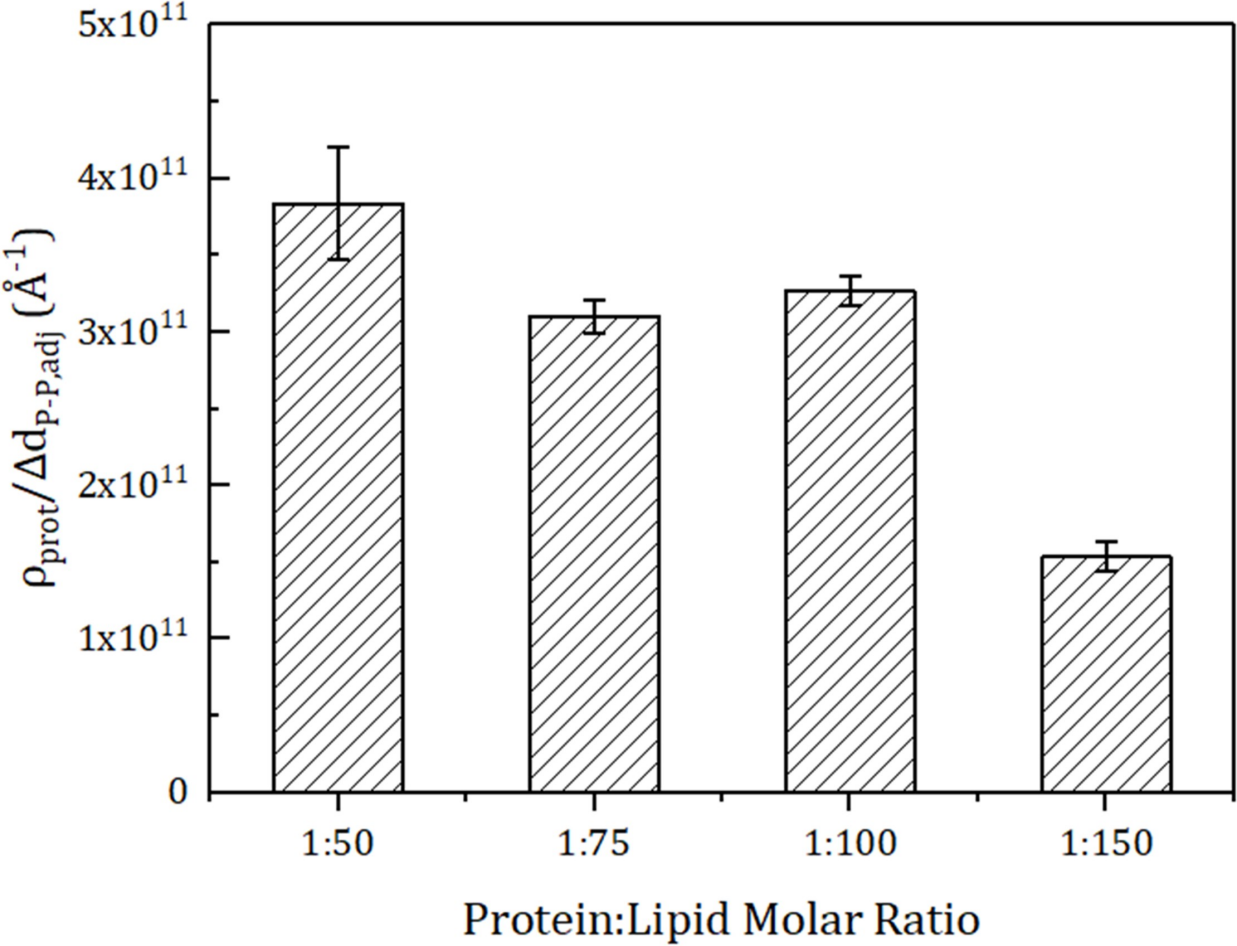
Prediction of the tendency of osmotic permeability for an aquaporin-lipid tetramer system (*p*_*f*_). The amount of aquaporin in the unit area (protein density, ρ_prot_) is calculated with a unit area of 1 cm^2^ and is proportional to *p*_*f*_ (*p*_*f*_ = ρ_prot_∙*p*_*u*,*tet*_). Therefore, *p*_*f*_ is predicted from Δd_P−P,adj_ and ρ_prot_ (*p*_*f*_ ∝ ρ_prot_/Δd_P−P,adj_). The predicted tendency of the *p*_*f*_ is highest at the protein-lipid molar ratio of 1:50, and followed by 1:100, 1:75, and 1:150. The error bars indicate standard errors.

Next, we predicted the optimal molar ratio condition for the osmotic permeability of the membrane system of the unit area (*p*_*f*_) with the structural parameter, protein density (ρ_prot_). Here, ρ_prot_ represents the number of channel proteins in the unit area of 1cm^2^. Because *p*_*f*_ is proportional to *p*_*u*,*tet*_ and ρ_prot_, the *p*_*f*_ trend can be derived from the formula ρ_prot_/Δd_P−P,adj_ (Fig 6). The protein-lipid molar ratio of 1:50 has the highest value of *p*_*f*_, followed by the molar ratio of 1:100, but the 1:100 case is advantageous in terms of cost and stability. When using the same size liposome, the system with the molar ratio of 1:50 requires 62.6% more aquaporin than the system with molar ratio of 1:100, but has only 17.4% better permeability. Therefore, it can be judged that the case of protein-lipid molar ratio 1:100 is more economical. Moreover, the high density of the membrane protein makes the membrane unstable. The system with protein-lipid molar ratio 1:50 is mechanically more unstable than the case with 1:100, and it is not appropriate to use a mechanically unstable system for filtration. Also, since the limit protein-lipid molar ratio for forming an aquaporin-lipid vesicle is 1:40, one cannot achieve high permeability by just adding more aquaporin than the 1:50 case. Therefore, the protein-lipid molar ratio of 1:100 is the optimal case for making aquaporin Z-lipid membrane (POPC:POPG) filtration system.

### Dynamics of the channel and permeability

**Fig 7:**
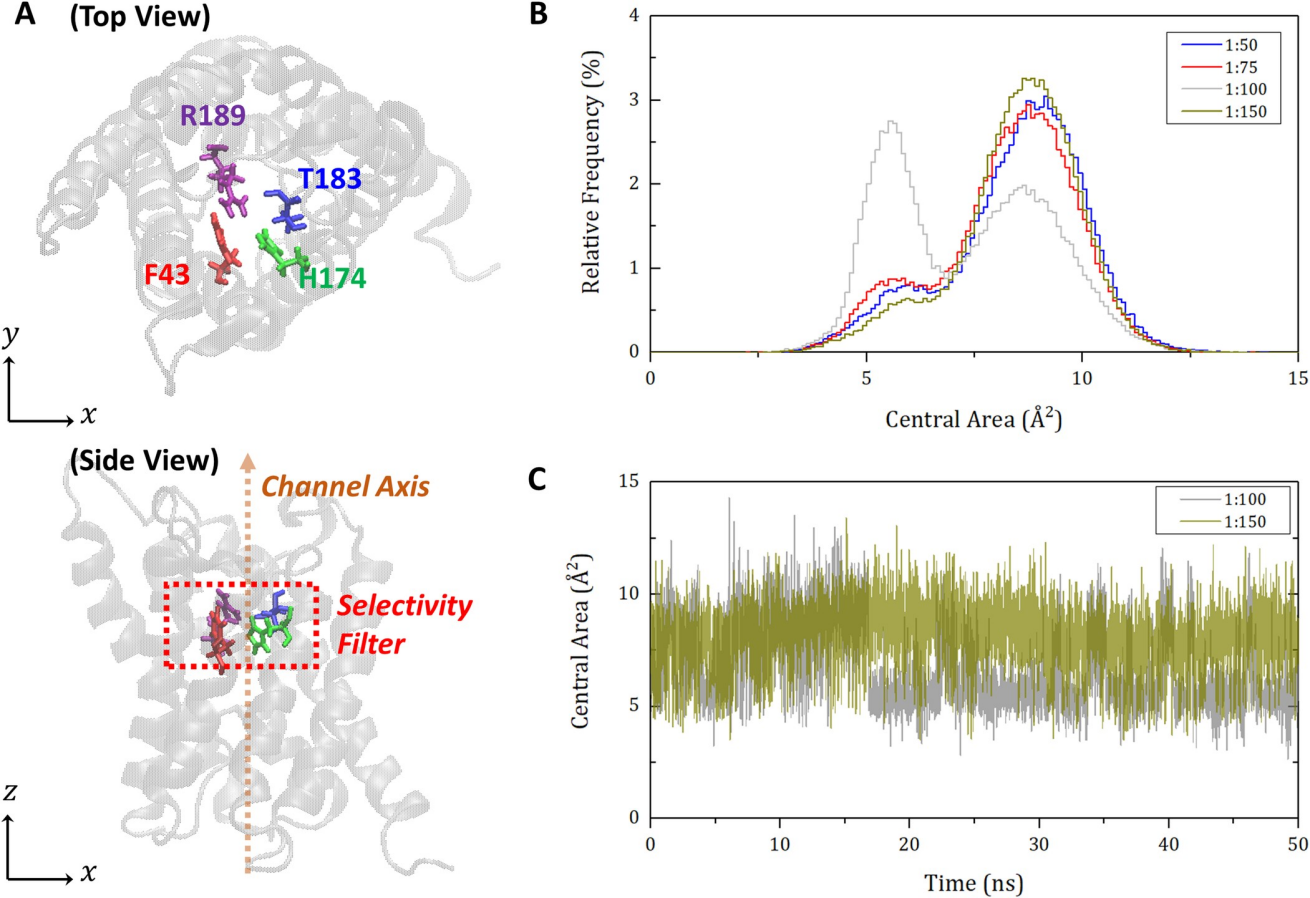
Schematic representation of the selectivity filter region of aquaporin and a relative frequency histogram of central area of selectivity filter. (A) Illustration of the selectivity filter region of aquaporin Z monomer in both top and side views. Selectivity filter region of aquaporin Z is defined as the region surrounded by four residues of F43, H174, T183, and R189. (B) Relative frequency histogram of central area of selectivity filter region for four cases of protein-lipid molar ratio. In our study, the position of an atom closest to the center point of four residues of selectivity filter region was extracted for each residue, and the area formed by the four points in the plane perpendicular to the channel axis was defined as the central area. Then, the central area of aquaporin Z monomer was plotted as a relative density histogram from the simulation data of 50 ns repeated four times for four protein-lipid molar ratios. (C) Variation of the central area of selectivity filter in the time domain. Central area changes of a monomer in the case of protein-lipid molar ratio 1:100 and 1:150 are plotted over the time.

To testify the correlation between hydrophobic mismatch and protein function, the change of aquaporin pore structures is observed over time. As shown in Fig 7A, the selectivity filter region of aquaporin Z is defined by the four residues of F43, H174, T183, and R189. Then, the area of the quadrilateral formed by the position of the four representative atoms on the plane perpendicular to the channel is calculated and called the central area of selectivity filter. This area data for 50 ns are extracted from each molar ratio simulation and plotted as relative frequency histograms in Fig 7B. Remarkably, the case of 1:100 fluctuates between two peaks around 5 Å^2^ and 9 Å^2^, while all other cases of 1:50, 1:75, and 1:150 mainly remain at a peak of 9 Å^2^. To visualize this fluctuation, Fig 7C compares the cases of 1:100 and 1:150 in the time domain. This indicates that the case of 1:100 is much more flexible (i.e., dynamical) than the others. This fact also supports our hypothesis such that the lower hydrophobic mismatch is much more favorable to the native resulting in the best function of proteins. That is, more natural, flexible, and dynamical movement of the selectivity filter helps water molecules pumped in and out of the channel. Consequently, the hydrophobic mismatch of aquaporin-lipid membrane system is associated with the protein-lipid molar ratio and it changes the channel stiffness resulting in water permeability.

## Conclusion

**Fig 8:**
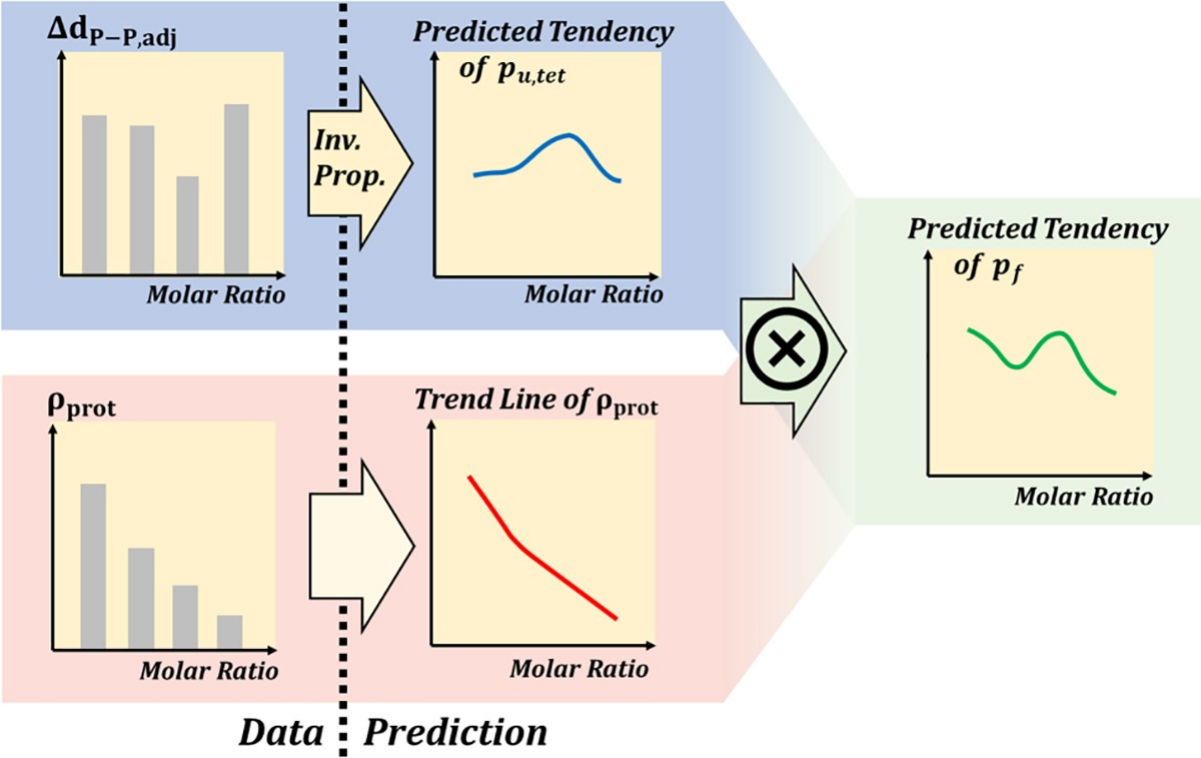
Schematic of the trend prediction of aquaporin permeability with a short production simulation of 5 ns. Data such as Δd_P−P,adj_ and ρ_prot_ can be obtained by short production simulation and are required to predict p_u,tet_ and *p*_*f*_, whose calculations using the collective coordinate model [27] need production simulation of more than 50 ns.

We investigated the importance of the aquaporin-to-lipid molar ratio in a aquaporin-lipid membrane system by calculating the *p*_*u*,*tet*_ value, and verified our results with structural parameters (Δd_P−P,adj_ and ρ_prot_) derived from a short production simulation of 5 ns. We also proposed a prediction method for *p*_*u*,*tet*_ with those structural parameters. As shown in Fig 8, Δd_P−P,adj_ represents the degree of stress the aquaporin receives, so a *p*_*u*,*tet*_ trend can be predicted by 1/Δd_P−P,adj_. In addition, the osmotic permeability of the membrane system of the unit area (*p*_*f*_) is proportional to the product of *p*_*u*,*tet*_ and the system protein density ρ_prot_, as expressed as ρ_prot_/Δd_P−P,adj_. By following this process, we could find the optimal conditions for the unit tetramer permeability and system permeability with Δd_P−P,adj_ and ρ_prot_. Although measuring the permeability directly from the long simulation can found the optimal condition more accurately, indirectly predicting the permeability using these structural parameters has an advantage in terms of time. If we want to explore the optimal protein-lipid molar ratio for various aquaporin types and various lipid compositions in order to design a real aquaporin-lipid membrane filtration system, quick prediction using Δd_P−P,adj_ and ρ_prot_ presented in this study will be a good tool in the early process of design. This is because it is difficult in terms of cost and time to make and check a real aquaporin-lipid membrane system for all conditions, and calculating permeability directly using simulation takes very long time and high computing resources to find the optimal condition. As suggested in this study, using a method of predicting the trend of permeability using Δd_P−P,adj_ and ρ_prot_ is much less costly than building a real system and enable us to derive optimal conditions by using only about 1/10 of the simulation time required for direct permeability calculation.

In summary, our methodology provides in-depth insights into the prediction of optimal conditions for permeability and enable more constructive and efficient design in future experiment or filtration system design. First, by using structural parameters that can be obtained from a short production simulation, it overcomes the long production simulation time of calculating water permeability. Second, it suggests how important the change of the lipid membrane adjacent to aquaporin is to determining water permeability, and it improves existing concepts for designing an ideal membrane system to achieve optimal permeability.
